# Clinics

**Published:** 1883-11

**Authors:** J. A. Robinson

**Affiliations:** Jackson, Mich.


### Clinics.

BY J. A. ROBINSON, D.D.S., JACKSON, MICH.

A clinic is a discourse delivered for practical instruction in presence of those who may be inexperienced, or who have had less experience; and so while the clinic is being performed the operator is expected to advise and give reasons for such advice; and lecture and talk during the operation. It is not easy to come to the best results by the study of any one plan, or any one system, and it is clearly unsafe to follow any one plan, and call it the best without we have carefully studied all other plans. It is not as important for the dentist to know that one eighth of an ounce of Gold foil can be packed in a single hour with some pet machine, as it is that he should thoroughly understand the best way to prepare the cavity to hold the gold securely after it is packed; and the best way to seal the margins so it will do good service when it is finished, and this can only be done by the omnipotant power of talk and illustration during the operation, to command the attention, and give inspiration and confidence to those looking on while the clinic is going forward. To simply perform an operation without a discourse, or passing from premises to consequences would be like the children before the blackboard in the school-room, pointing out a place on a map without talking of the boundaries, or solving a problem in mathematics without giving the rule for doing the sum. As the unit of numbers is *one*, and the unit of the mechanical powers is the lever; so the unit of the senses is the understanding, and it is by the understanding, and skill to enforce the understanding; that we are able to become dentists. Without this we are like the boys in the school-room, who copy their problems from the slates of those who are up in the class, and so pass examination on the merits of those who have understanding enough to accomplish the work. Filling and preserving the teeth is also a question of morals as well as of mechanics, for no person is fit to practice dentistry, who does not realize the value of the organs, and the *man;* and has faith that he is able to do the work in such a manner as will accomplish the object.

With this preliminary discourse on the importance of the work before us, and with the rubber dam well in place, we will proceed with the second molar, on the right side of the lower jaw on the posterior surface. The cavity is near the gum, and is a fissure that extends nearly the whole length of the back side of the tooth. A dish-shaped corundum disk will separate it sufficiently to operate, and as the tooth back of this is sound, and is a wisdom tooth, it is better that the enamel should not be disturbed. After it is sufficiently separated, commence at the center of the tooth at the top, and cut down vertically till we meet the cavity. This will give a cavity the shape of an inverted T. The direction of the burr must now be changed, (it ought to be a *stoned-burr,*) and run very fast with a heavy hand piece to prevent stuttering or chattering, and run in a direct line from the top to the bottom till it is the shape of an inverted V, and cut until you can see every part of the cavity, and deep enough to form a good anchorage on either side. A spoon-shaped excavator, somewhat like a long handled shovel, will fit the cervical wall, and leave a little trough longitudinally that must be thoroughly cleansed of all decayed substance, particularly at the corners.

The buccal and lingual corners will be the most difficult to secure, and they must be filled first. An orange-wood wedge, running below the line of decay, and half way to the top of the tooth, for a matrix is necessary to keep the first pellets in position, and is better than retaining-points. Now, cut from a leaf of No. 4 gold foil a strip seven-eighths of an inch wide, and divide it in the middle; crump it into four or five folds with a knife while it lays on the fore-finger of the left hand; turn all the edges in, and double it in the middle, so it will be about three-eighths of an inch long, and give it a slight roll with the thumb and fore-finger. If you cut another strip across the short side of the gold, and then another, you will find the balance a little more than a quarter of the size when you begin, then make the remainder into small pellets, and we will place them in the cavity. Take the two largest ones and anneal the *butt-end*, and place them the soft end down in the corners of the cavity at the bottom (I am supposing this is Williams' or some corrugated foil, which is a soft foil, but is cohesive when heated), and drive them well home into the corners of the cavity, and be certain they are in place and tight, then take another large pellet and place it between the corner pellets, and make a tight bridge across the cervical wall, the center will wedge the two corner pellets first introduced. The orange-wood wedge will give a little, and allow the gold to come flush with the outside of the cavity. It is a fundamental principle that no filling should project at the borders beyond the enamel in any portion of the tooth. The object of annealing the pellet at the *butt-end* is, that it is harder when annealed, and serves as a sort of head to force the soft gold into place, and at the same time makes it cohesive to receive the soft end of the next pellet that follows it. A cork-screw serrated plugger that is stoned so that it will not load, or pull back the gold is best for the work. What I mean by a cork-screw plugger is a plugger that is twisted half round like a ram's horn, so that the shank follows the point to give a direct blow, and it can be used equally well in any direction.

There are plenty of pluggers made similar to the one I have described. You will find them in the "Watling set." They do well for hand pressure but are out of line with the shank, and uncertain when struck with the mallet.

This process of filling across the cavity from the buccal to lingual surface must be repeated till you are within a line from the top or outcome, and the balance perhaps had better be filled by hand pressure with cohesive foil and finished with two or three layers of No. 60. This clinic has been performed entirely with gold foil, but many of our best operators think a cavity of this kind will serve a better purpose that is filled within a line of the top with Robinson's fibrous and metallic filling, and finished with gold, than can be made with gold foil however carefully done. The fibrous filling is the softest metallic filling known to the profession, and being fibrous recoils *within itself* instead of *on itself*. The matter of contour and finish must be left to the individual judgment and taste. It is the *best way to preserve the teeth* and to make them useful that is interesting us at this time.

Our next operation will be to take care of this left front incisor that has been broken off, commencing at the mesial line and running at an angle of 45 degrees posteriorly so that one-third of the tooth is gone. The dentine has turned dark but the tooth is still alive. It will be necessary to put a screw into the tooth after it is excavated in a suitable manner to sustain a filling. This screw is made of platinum wire about 23 of the gauge plate. It is not cut in a screw plate but wound with gold foil No. 4 and held in the gas jet until it is melted and plated with gold. Now take some platinum binding-wire No. 36 and hold it at right angles at the end of each piece of wire and it will unite or solder to the gold surroundings the 23 wire—twist the fine wire around the large wire in the form of a screw and wind the screw thus made with gold foil, heat it till the foil is melted and you have a platinum gold-plated screw that is better than one made of solid gold, because the thread is coarser and it is stiffer than pure gold. Drill your hole as near the palatine surface as you can safely with a drill wet with glycerine deep enough to take five or six turns of the screw and cement it in place with "Weston's Insoluble Cement." We will commence this cavity with the pellets heated at the *butt-end* and pack the soft end at the base and around the screw, and let the *butt-ends* project a little beyond the enamel of the tooth. Make the base after the cavity is evened up with the gold as level as possible but packing the margins with the *butt-ends* of the pellets, keeping the soft foil in the center of the cavity. When you have one-fourth of the space filled that you wish to restore, it will present the appearance of the leaves of a flower in blossom. These ends must now be consolidated to overlap the margin of the enamel enough to finish. Take some cohesive foil and roll into rope about the size of the pellets of commerce, and cut into lengths about one-half an inch long, and pack them, on the margins of the gold in the tooth half the length of them and then turn the end across the center of the filling and weld them on. This must be repeated until the contour is nearly restored and then the whole plated with No. 60 cohesive foil, and even up all depressions with pellets. Now add cohesive foil cut a little larger than the gold filling, and turn back the edges that project over so to keep the edges square and fit to finish.

The consolidation should be done with a serrated foot plugger slightly oval on the face to avoid the possibility of checking the borders of the enamel. Another point gained, by using a plugger of this sort, is that every blow expands the gold while it welds it. The whole should be faced with heavy gold beat on with a smooth oval-faced oblong foot plugger, *a planisher* that is more sure to make it solid and gives a better finish.

An instrument of this shape is the best for amalgam fillings. All melted metals assume a globular or spherical form, and the globules of mercury, however small, correspond to metals in a state of fusion. The silver when combined with mercury and dissolved is in the effort to become a sphere, so all attempts to consolidate amalgam fillings with a mallet after they are placed in the cavity only serves to draw them away from the margins of the cavity, and to disintegrate the mass into globules that will admit air and moisture while an oval faced plugger will expand and flatten the mass, and leave it at the borders as perfect as is possible with this imperfect but sometimes useful filling. For the various plastic filling materials I refer the reader to some experiments in the *Independent Practitioner* of August, 1883, on page 417. If this clinic is somewhat long you must remember that it is not half as long as you would stand by the chair and see the work done, and it will probably pay some who are inexperienced to read it with care. Clinics should be performed in a missionary spirit. It would be so easy for those of twenty-five years experience to help the young over some of the difficulties that once caused them trouble and now seem so plain. It would do the world more good than anatomy or physiology or histology or the pyorrhæ alveolaris craze that seems to have taken possession of the whole profession.

